# Exploring the Diagnostic Capability of Artificial Intelligence in Dermatology for Darker Skin Tones: A Narrative Review

**DOI:** 10.7759/cureus.94909

**Published:** 2025-10-19

**Authors:** Teniola Dowie

**Affiliations:** 1 Accident and Emergency, Northern Care Alliance Trust, Manchester, GBR; 2 Accident and Emergency, Fairfield General Hospital, Bury, GBR

**Keywords:** artificial intelligence in medicine, cutaneous oncology, dark skin, dermatopathology, diverse populations, medical dermatology

## Abstract

As the use of artificial intelligence (AI) as a diagnostic tool increases in the field of dermatology, there has been a growing need to diversify datasets to improve its diagnostic capability in darker skin tones. Currently, AI is not as effective as a diagnostic tool in darker skin tones (Fitzpatrick IV-VI) as it has been in lighter skin-toned (Fitzpatrick I-III) populations. This narrative review will provide a summary of the recent data and advancements made within the area. Medline and PubMed databases were searched with the following search terms: dermato* AND (skin tone or race or skin colour or ethnicity or race or Fitzpatrick) AND (ai or artificial intelligence). Texts were filtered for full text and English language from 2020 to 2025. Results including patients under 18 years of age were excluded, which resulted in 52 papers. After scanning through titles and abstracts, a total of eight papers remained that were relevant to the review. AI models have demonstrated lower accuracy in recognising cutaneous pathology in darker skin tones in the majority of studies. When looking at the results after training the models with diverse datasets, there was an overall improvement in the accuracy of AI to recognise pathology in Fitzpatrick skin tone IV-VI. Several studies also showed that there is some benefit to training AI with artificially pigmented images to improve its accuracy. AI has significant potential to enhance dermatology by improving diagnostic accuracy, reducing variability, and improving efficiency. Expanding datasets further appears to be of benefit in improving accuracy in darker skin tones. Further studies with larger sample sizes are needed to analyse other reasons the algorithms have lower accuracy in darker skin tones and how this could be mitigated.

## Introduction and background

Artificial intelligence (AI) has been increasingly used within the field of dermatology for diagnostic purposes. AI models have been developed to recognise and flag malignant and non-malignant lesions after being trained with databases containing image samples from patients with a range of dermatological conditions [1]. The models have shown potential to assist with the diagnosis and treatment of a variety of cutaneous pathologies [1]. Along with their utility, there has been a growing need for the expansion of the datasets to improve their accuracy in darker-skinned patients [2]. As it stands, AI models have largely been trained on populations with lighter skin tones [2]. Patients with lighter skin tend to present more frequently than patients with darker skin for conditions such as melanoma; this could potentially contribute to lower representation [3]. Due to a lack of diversity in the training sets, AI has shown difficulty in accurately recognising pathology in darker skin tones [4]. Many studies lack skin tone data and therefore cannot accurately assess the efficacy of AI on varying skin tones [5]. This narrative review will explore the accuracy of dermatological AI in Fitzpatrick skin tones IV-VI compared to I-III, limitations of AI, and solutions for how algorithms may be improved in the future.

**Figure 1:**
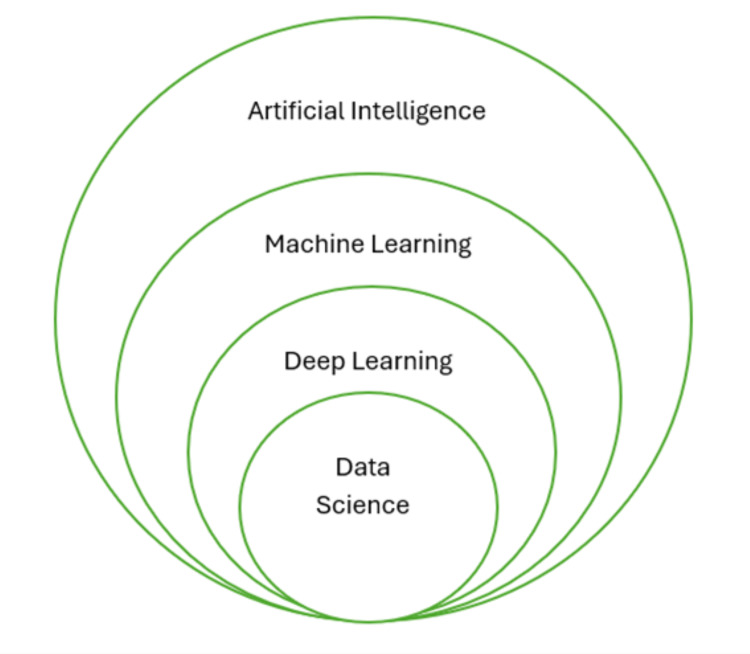
Diagram of the classification of artificial intelligence subsets Image created by the author.

AI eases tasks that typically require human intelligence to be carried out sufficiently using techniques to mimic or replicate human intelligence [6]. It encompasses various subsets, which include deep learning (DL) and machine learning (ML) (Figure 1) [6,7]. ML is a branch of AI that focuses on creating algorithms that learn from data to then improve themselves without explicit supervision [7]. DL is a branch of ML that uses neural networks, which are modelled after the human brain [6]. They can process large amounts of complex data and have shown potential to be a great diagnostic tool for skin diseases [6,7].

Early Use of AI in Dermatology

The use of AI in the field of dermatology was first described in 1987 with the interactive, text-based computer program TEGUMENT [8]. This allowed dermatologists to compare specimens with a structured knowledge base on a computer until they reached a diagnosis [8,9]. These early iterations of AI have paved the way for the more recent, autonomous algorithms used within medicine, such as the Deep Ensemble for Recognition of Malignancy (DERM) model [10]. AI models such as DERM have recently been used as a diagnostic aid to determine the chance of malignancy in cutaneous lesions [1,10]. Current AI models have largely been trained on lighter-skinned populations; this has contributed to a weaker diagnostic capability when used on darker-skinned patients [11]. There has been a need to diversify training images to improve the accuracy and better serve a global population [11].

Current Use of AI in Dermatology Triage

AI has increasingly been used in tele-dermatology and clinics for triage purposes [12]. Patients can send in images for review or be assessed in person with a dermoscopic lens attached to a smartphone for a higher-quality image [13]. An algorithm is then used to triage these lesions to determine if they need to be referred to a dermatologist [13]. Several studies, including a systematic review published in 2024 looking at AI use in dermatology, suggest the AI models may perform to a lower standard in darker skin as they are not trained on diverse datasets [4]. One reason that datasets may lack diversity is due to populations with darker skin showing lower rates of developing skin cancers such as melanoma [3]. Although they make up a smaller proportion of skin cancers, darker-skinned patients often have a higher mortality rate due to a late presentation with the disease [14]. A lack of diversity in the AI training sets could result in delayed or incorrect diagnosis and potentially widen health disparities, if not further developed [15]. This emphasises the importance of improving early diagnosis and accurate triage for darker-skinned patients.

Use of AI for Diagnosis of Cutaneous Malignancy

AI has shown efficacy in the recognition of malignant skin lesions, such as melanomas and basal cell carcinomas (BCC) [16]. A systematic review in 2023 showed that, from the 232 studies reviewed, the overall accuracy, sensitivity, and specificity of AI for the detection of cutaneous malignancy averaged 90%, 87%, and 91%, respectively [16]. An issue highlighted in the study was that, out of all the studies included in the review, only 1.3% described the Fitzpatrick skin type and only 3.2% of the images were type IV-VI [16]. As AI builds on its diagnostic capabilities through the datasets that it is trained on, there is a need to diversify training sets to produce higher diagnostic accuracy on future specimens [15]. This narrative review will provide a summary of the recent data and advancements made within the area.

## Review

Methods

A literature search was conducted in September 2025 using the databases for Medline and PubMed to identify relevant literature. The search focused on studies published between January 2020 and September 2025 to build on previous reviews. The focus was on papers that discussed the efficacy of AI in darker skin tones and solutions to improve this. Each study included in the review had to show representation of Fitzpatrick skin types IV-VI. As this is a narrative review, meta-analysis and assessment of bias were not performed. The following search terms were used: dermatolo* AND (skin tone or race or skin colour or ethnicity or race or Fitzpatrick) AND (ai or artificial intelligence).

The following filters: full text and English language were also applied. Studies that included paediatric patients were excluded. The PICO framework has been included in Table 1.

**Table 1:** PICO framework PICO: Patient/Problem, Intervention, Comparison, and Outcome

PICO Elements	Inclusion	Exclusion
Population	Studies including adults	Studies including participants under the age of 18
Intervention	Use of artificial intelligence to diagnose dermatological pathology	Non- dermatological conditions
Comparison	Comparing efficacy of AI with light (I-III) and darker (IV-VI) skin tones on Fitzpatrick scale	No comparison for different skin tones
Outcome	The accuracy of AI in different skin tones	No outcome measures related to skin tone

Results

Following this search, 25 papers from Medline and 28 papers from PubMed were identified. Out of these 53 papers, 12 were duplicates. The remaining 41 papers were screened for relevancy within their titles, abstracts, and full-text papers. Any systematic or narrative reviews were removed. Following this process, eight papers remained and were analysed for this review. The summary of these can be seen in Table 2.

**Table 2:** Summary of the final papers included in the review

1^st^ Author/Publication date	Title	Objective	Findings
Benčević et al. 2024 [17]	Understanding skin colour bias in deep learning-based skin lesion segmentation	Evaluate skin tone bias within AI models for skin lesion segmentation in data sets PH2 (200 images), Waterloo (191 images) and Dermofit (1300 images)	A large correlation between segmentation performance and skin colour, with bias against darker skin tones
Aggarwal 2021 [18]	Performance of artificial intelligence imaging models in detecting dermatological manifestations in higher Fitzpatrick skin colour classifications	Assess the image recognition performance of dermatological diseases. Data set 1 with type I-III and data set 2 with type IV-VI skin tones. Each set with 150 images of confirmed dermatological malignancy (BCC and melanoma)	AI models trained on images with lighter skin colour had higher sensitivity, specificity, positive predictive value and negative predictive value than the image recognition models trained on darker skin tones for differentiation between melanoma and basal cell carcinoma
Patel Housley et al. 2025 [19]	Performance evaluation of ChatGPT-4o in dermatological diagnoses across Fitzpatrick skin types	Evaluated ChatGPT-4o’s diagnostic performance across Fitzpatrick skin types using 324 dermatologic images that have been confirmed via biopsy	Results showed significantly lower sensitivity, specificity, and accuracy for melanoma in darker skin tones (FSTs III-VI) compared to lighter tones (FSTs I-II)
Rezk et al. 2022 [20]	Leveraging artificial intelligence to improve the diversity of dermatological skin colour pathology: protocol for an algorithm development and validation study	Develop and evaluate an AI model for an early detection system for all skin tones using clinical images. Images were artificially generated to increase the diversity of the datasets and the effects of these were analysed	AI diagnostic ability was tested before and after being trained with a diverse data set. Accuracy increased from 0.88 to 0.94. Sensitivity increased from 0.72 to 0.73. Specificity significantly increased from 0.91 to 0.98
Daneshjou et al. 2022 [21]	Disparities in dermatology AI performance on a diverse, curated clinical image set	‘Diverse Dermatology Images (DDI)’ dataset created to assess the performance of current AI models: ModelDerm, DeepDerm, and HAM10000 on diverse skin tones	AI models assessed exhibit limitations on the DDI dataset, particularly on dark skin tones and uncommon diseases
Schneider et al. 2023 [22]	Diagnosis of skin disease in moderately to highly pigmented skin by artificial intelligence	Determine the performance of the Triage Inc AI model in the screening and triage of benign-neoplastic, malignant, neoplastic, and non-neoplastic skin conditions for Fitzpatrick IV-VI skin types	163 images of skin disease manifestations from Fitzpatrick IV-VI. All photos were primarily diagnosed by a specialist and AI was then compared to this baseline. AI had an overall accuracy of 86.50% in diagnosing skin disease in Fitzpatrick IV-VI skin types
Aggarwal et al. 2022 [23]	Artificial intelligence image recognition of melanoma and basal cell carcinoma in racially diverse populations	Improve the performance of AI models in recognizing cutaneous diseases in individuals with darker skin tone by artificially darkening dermatological images from lighter-skinned samples	Training AI on artificially “darkened” images resulted in a higher sensitivity, specificity, positive predictive value, negative predictive value and F1 score compared to training on the original “light” images
Kamulegeya et al. 2023 [24]	Using artificial intelligence on dermatology conditions in Uganda: a case for diversity in training datasets for machine learning	Assess the diagnostic performance of the AI-powered dermatological algorithm ‘Skin Image Search’ on Fitzpatrick VI skin	Overall diagnostic accuracy of the AI model on Type VI skin was 17% compared to 69.9% performance on light skin tones as reported from the training results

Discussion

This review will focus on three main themes found in the literature: the current diagnostic capability of AI models on darker skin, the limitations of AI models, and the possible solutions that could be implemented.

Diagnostic Capability of AI on Darker Skin

Overall, the literature has shown that AI is less accurate for darker skin tones. The study by Benčević et al. used the Dice Similarity Coefficient (DSC) to provide a measure of sensitivity and precision for evaluation [17]. Using Spearman's rank correlation, the study found a significant negative correlation between DSC and p(FP=V-VI), showing that darker skin tones had less accurate and less precise results due to difficulty with segmentation [17]. In Aggarwal's study, the AI model had an accuracy of 60% at picking up melanoma in the lighter skin dataset compared to 53% in the darker skin dataset [18]. Conversely, Schneider et al.'s study showed that AI had an overall accuracy of 86.5% in diagnosing skin disease in Fitzpatrick IV-VI skin types [22]. While the study showed a high accuracy in darker skin compared to other papers, they note that malignant disease had low representation (75% non-neoplastic, 14% neoplastic-benign, 11% neoplastic-malignant) [22]. Aggarwal et al. have also shown an accuracy drop due to the lack of diverse images in datasets [23].

A study by Patel Housley et al. using ChatGPT-4o demonstrated a significantly lower sensitivity, specificity, and accuracy for melanoma diagnosis in individuals with darker skin tones [19]. For melanoma, skin types I-II had a sensitivity of 100% ± (95%, 57-100%), type III-IV 29% ± (95%, 8-64%), and V-VI 43% ± (95%, 16-75%) [19]. Additionally, Fitzpatrick types I-II showed an accuracy of 71% ± (95%, 62-78%), while types V-VI had 42% ± (95%, 30-54%) for detecting melanoma [19]. Kamulegeya et al.'s study showed an overall diagnostic accuracy of 17% for the skin image search AI model for type VI skin [24]. This was compared to 69.9% for lighter skin types as reported from the training results [24]. Although overall accuracy was 17%, the model showed an 80% accuracy in specific conditions, such as dermatitis, compared to 0% in fungal infections, showing that the capability varies depending on the pathology [24].

Limitations of AI

Darker skin tones have been underrepresented in datasets, meaning that AI has not been adequately trained to recognise disease in these patients. The improvement of the AI models would require an expansion of these datasets to make them more diverse and increase accuracy. The study by Aggarwal noted difficulty due to limited images for type IV and V skin tones, which meant that the study had to be scaled down [18]. Despite having the same proportion of images in the datasets for darker skin (IV-VI) and lighter skin (I-III), the lighter skin dataset still achieved a higher sensitivity [18]. This suggests that perhaps a greater amount of data is needed to train the AI algorithm to identify pathology in darker skin as accurately as it is able to in lighter skin. Daneshjou et al.'s study describes the issue of label noise within the datasets, reducing accuracy [21]. To create datasets, images are viewed by dermatologists and labelled (e.g., malignant or non-malignant) [21]. There is often no follow-up for these images to confirm if these samples are malignant following biopsy; therefore, they remain labelled incorrectly [21]. The low diversity of datasets, paired with the low or non-existent number of confirmed pathologies in darker-skinned samples, also contributes to the low accuracy of AI [21].

The low specificity seen in some models may be due to greater variability in the IV-VI skin types and difficulty in distinguishing the lesion from the surrounding skin [18]. Benčević et al. suggest that, as the contrast between the lesions and surrounding tissue is lower, lesions are harder to segment in type V-VI skin tones [17]. Kamulegeya et al. also highlight the need for high-quality images to train AI [24]. If the lighting and angle of the camera are not optimised, then the image sample may be compromised and would have to be excluded from the dataset. Patel Housley et al.'s study did not include any BCC in the V-VI skin types, and the ChatGPT-4o model had no context for the site of the lesion or the patient’s history, which may impede diagnostic capability [19].

Solutions to Improve the Diagnostic Ability of AI in Darker Skin

Multiple studies reiterate the need for high-quality, diverse datasets for the AI models to perform to a higher standard [18,24]. There is some difficulty in expanding datasets, as typically patients with higher Fitzpatrick skin type have lower rates of developing cutaneous malignancies [14]. Rezk et al. have attempted to expand datasets with high-quality artificially produced images of darker skin with dermatological pathology [20]. Initial tests on segmentation show high accuracy, and it may be a beneficial solution in the interim until datasets are expanded with real images [20]. Aggarwal et al.'s study similarly trained an AI model on artificially darkened images [23]. They found an increased sensitivity and specificity for the AI model in differentiating between BCC and melanoma in darker skin [23]. Daneshjou et al.'s study produced a dataset with real histopathological samples collected from patients with confirmed benign and non-benign lesions [21]. This resulted in an improvement in the performance of the AI on lighter and darker skin and helped close the gap in disparity [21].

Benčević et al. suggest a potential solution is to provide more descriptive annotations, rather than a binary of lesion vs non-lesion areas, expanding the labels to describe lesions in greater detail (i.e., white, yellow, black, and hypopigmented areas) [17]. This could help the AI models improve border contrasts, shapes, smoothness, and therefore enhance the accuracy of segmentation [17].

Limitations

There was a limited number of papers available that were relevant to the aim of this review. Many studies available had small sample sizes for darker-skinned images, which may also have produced less accurate data. There is a need for further studies on a larger scale to be developed in the future. These studies would need to include the comparison of AI on different Fitzpatrick types.

## Conclusions

Overall, the literature examined has shown that the accuracy of AI in detecting cutaneous pathology in darker skin tones is lower than it is in lighter skin. There is still a need for further research on effective methods to close the gap on the diagnostic capability of AI across Fitzpatrick skin types. Future studies would benefit from using larger samples sizes from populations with Fitzpatrick IV-VI skin tones to train AI models. As the number of images available is low, there may be a benefit to the use of artificially darkened images to train AI models in the interim, until a larger number of real images are collected. As well as expanding datasets to be more diverse, future studies would also benefit from training AI models to provide more descriptive annotations. This may help the models identify and distinguish pathological lesions in darker skin, where often there is difficulty due to lower contrast between the surrounding areas and the lesion. Further studies looking at the variability of lesions across skin types may also be beneficial.
